# A Case of Vogt-Koyanagi-Harada Disease: Diagnosis Based on the Presence of Vitiligo and Sunset Glow Fundus Without Ocular Symptoms

**DOI:** 10.7759/cureus.78209

**Published:** 2025-01-29

**Authors:** Honoka Tsutsui, Ken Fukuda, Isana Nakajima, Kenji Yamashiro

**Affiliations:** 1 Ophthalmology, Kochi Medical School, Nankoku, JPN

**Keywords:** autoimmune disease, sunset-growth fundus, uveitis, vertigo, vitiligo, vogt-koyanagi-harada disease

## Abstract

Vogt-Koyanagi-Harada disease is an autoimmune disease affecting melanocytes. We report a case of Vogt-Koyanagi-Harada disease where the diagnosis was based on the presence of vertigo, vitiligo, and sunset glow fundus without ocular symptoms. The patient was a 68-year-old woman with a history of type 1 diabetes mellitus who developed vertigo three months prior and vitiligo on the face and arms two months prior. An ophthalmic examination revealed sunset glow fundus with numerous depigmented chorioretinal scars. Keratic precipitates were observed; however, no cells were observed in the anterior chamber. The patient had been examined by an ophthalmologist five years prior for a thorough evaluation of diabetic retinopathy; at that time, the fundus was normal in color. Based on the vertigo, vitiligo, and fundus findings, the patient was diagnosed as having convalescent Vogt-Koyanagi-Harada disease. Physicians should be aware that Vogt-Koyanagi-Harada disease is a potential cause of systemic symptoms, including vertigo, hearing loss, headache, nausea, and vitiligo.

## Introduction

Vogt-Koyanagi-Harada (VKH) disease is a rare autoimmune disease affecting melanocytes that manifests as systemic inflammation in pigmented structures involving the eye, skin, and auditory, vestibular, and central nervous systems. Its manifestation differs clinically in early and late stages [1]. In the early stage, anterior uveitis and retinal detachment occur and are often recognized by ophthalmologists who become aware of vision loss or blurred vision.

In addition to ocular symptoms, extraocular symptoms occur in both the early and late stages [1-3]. In the early stage, patients may notice neurological symptoms such as headache and nausea due to meningitis and auditory symptoms such as tinnitus and hearing loss [4-6]. In the late stage, patients may present with sunset glow fundus, alopecia, poliosis, and vitiligo due to loss of pigmentation [7]. Complete or incomplete VKH is diagnosed by the presence or absence of the following five findings: 1) no history of penetrating ocular trauma or surgery preceding the initial onset of uveitis, 2) no clinical or laboratory evidence suggestive of other ocular disease entities, 3) bilateral ocular involvement (early or late manifestations), 4) neurological/auditory findings, 5) integumentary finding (not preceding onset of central nervous system or ocular disease) [8].

In this report, we describe a case of VKH based on the presence of extraocular symptoms, including vertigo and vitiligo, and sunset glow fundus without recognized ocular symptoms.

## Case presentation

**Figure 1:**
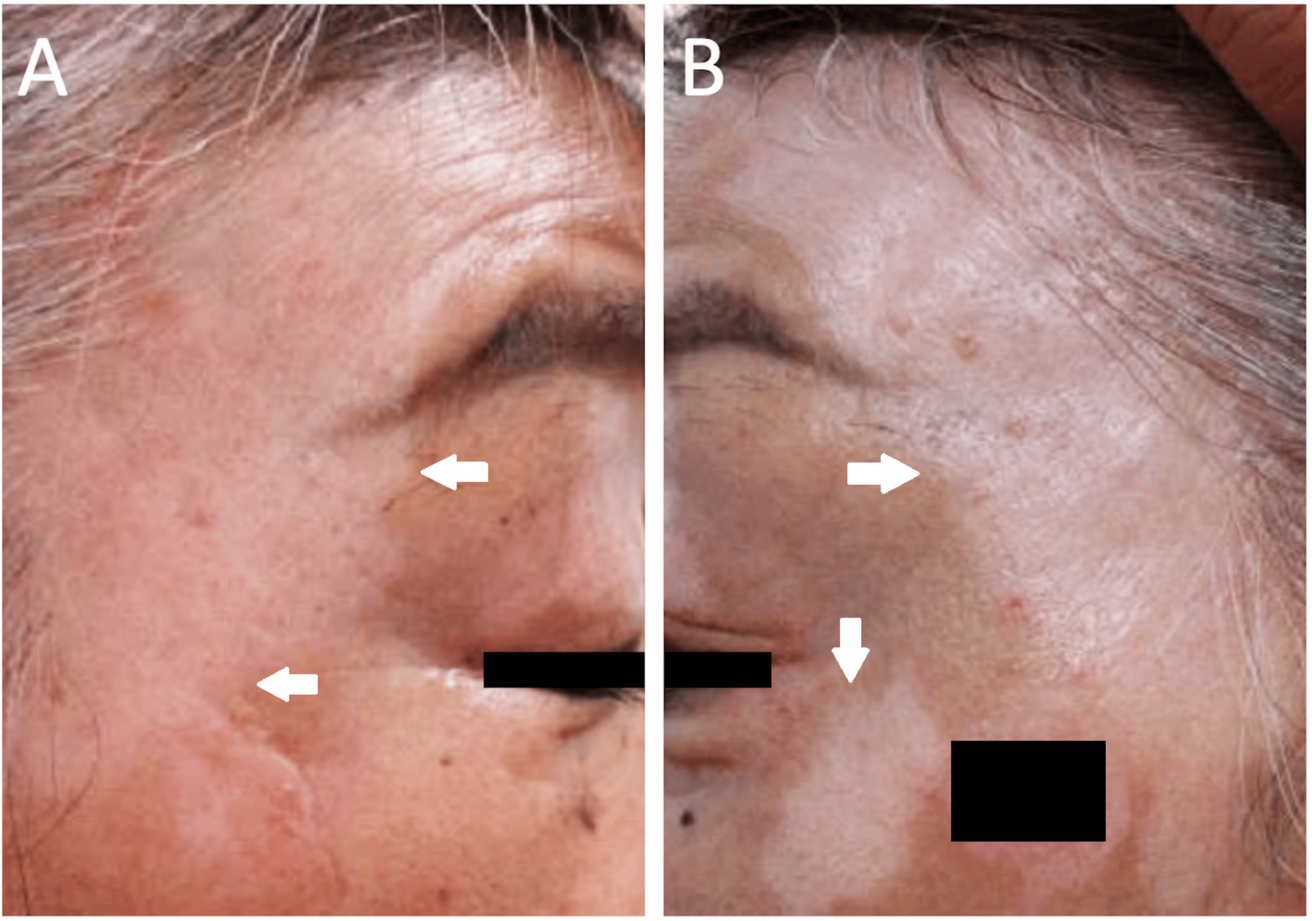
Photographs of the face Vitiligo (arrows) can be observed on the patient’s face (A, B).

A 68-year-old woman with type 1 diabetes was referred to the ophthalmology department after a dermatology evaluation of an unknown cause of vitiligo. The patient was referred from the dermatology department to ophthalmology for a thorough examination of Vogt-Koyanagi-Harada disease as a differential diagnosis of vitiligo. Three months before the current visit, the patient had experienced vertigo, abdominal pain, and difficulty standing. Neurological and otolaryngological examinations revealed vertigo and nystagmus of unknown cause. However, the vertigo did not improve. Vitiligo was present on the patient’s face and arm for two months (Figure 1).

A skin biopsy of the facial lesion revealed a decreased number of epidermal melanocytes. The patient did not use steroids or immunosuppressive medications for the systemic symptoms.

**Figure 2:**
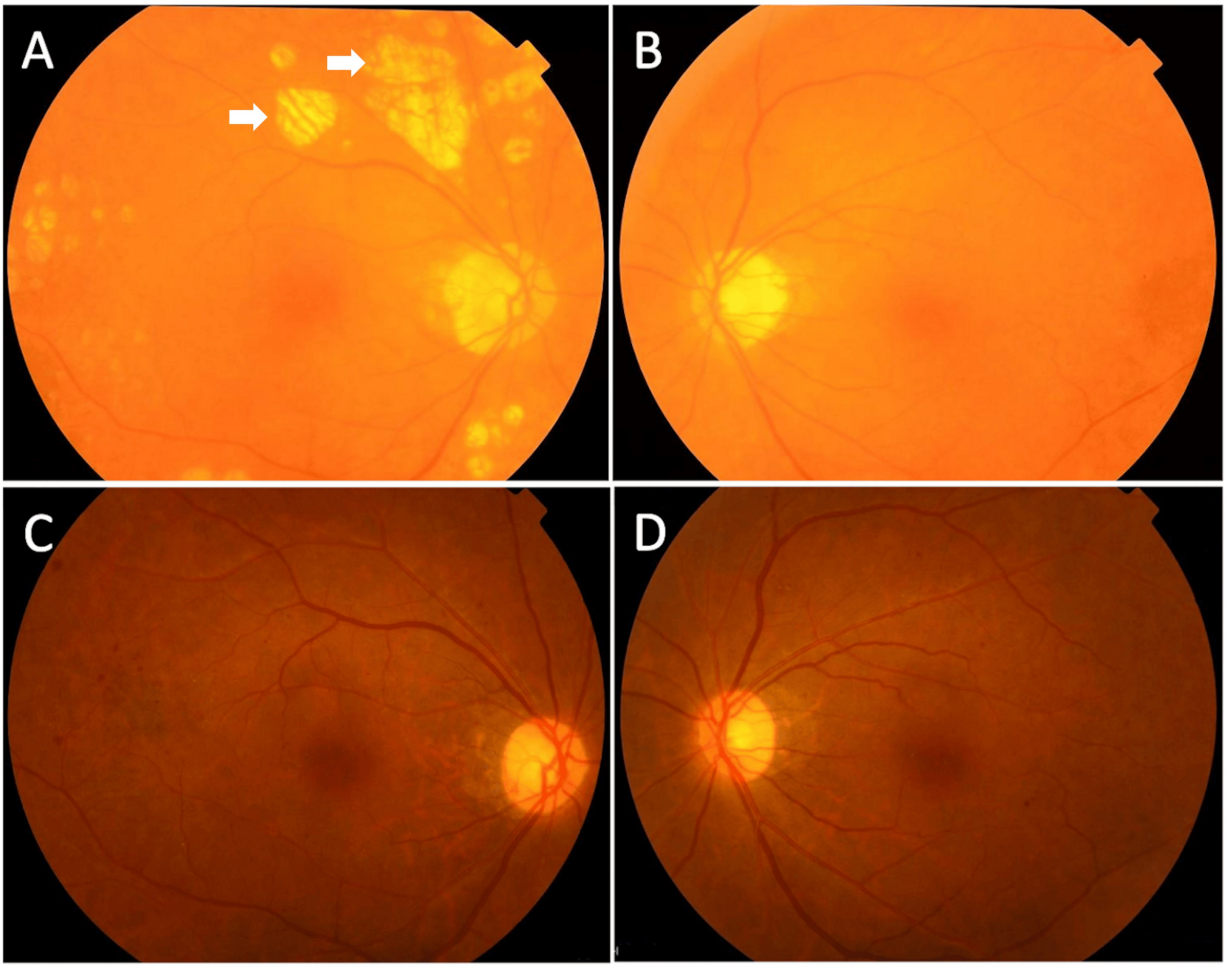
Photographs of the Fundus Photographs obtained at the current visit show bilateral bright red–orange choroids, representative of sunset glow fundus, with atrophic nummular hypopigmented lesions (arrows) at the current visit (A, B). Five years earlier, bilateral retinal hemorrhages were observed with a normal fundus color (C, D).

The best-corrected visual acuity was 20/25 and 20/20 in the right and left eyes, respectively. Intraocular pressure was 12 and 14 mmHg in the right and left eyes, respectively. Slit-lamp examination showed bilateral inactive pigmented keratic precipitates but no inflammatory cells in the anterior chamber. Bilateral pupils were round without synechiae. Anterior chamber depth was normal and cataract was also observed in both eyes. Fundus examinations detected bilateral bright red-orange choroids (choroidal depigmentation), representative of “sunset glow” fundus, with atrophic nummular hypopigmented lesions (Figure 2A, 2B). The macula appeared to be normal. Fundus examination performed five years ago had revealed retinal hemorrhages indicating diabetic retinopathy, with a normal fundus color (Figure 2C, 2D).

**Figure 3:**
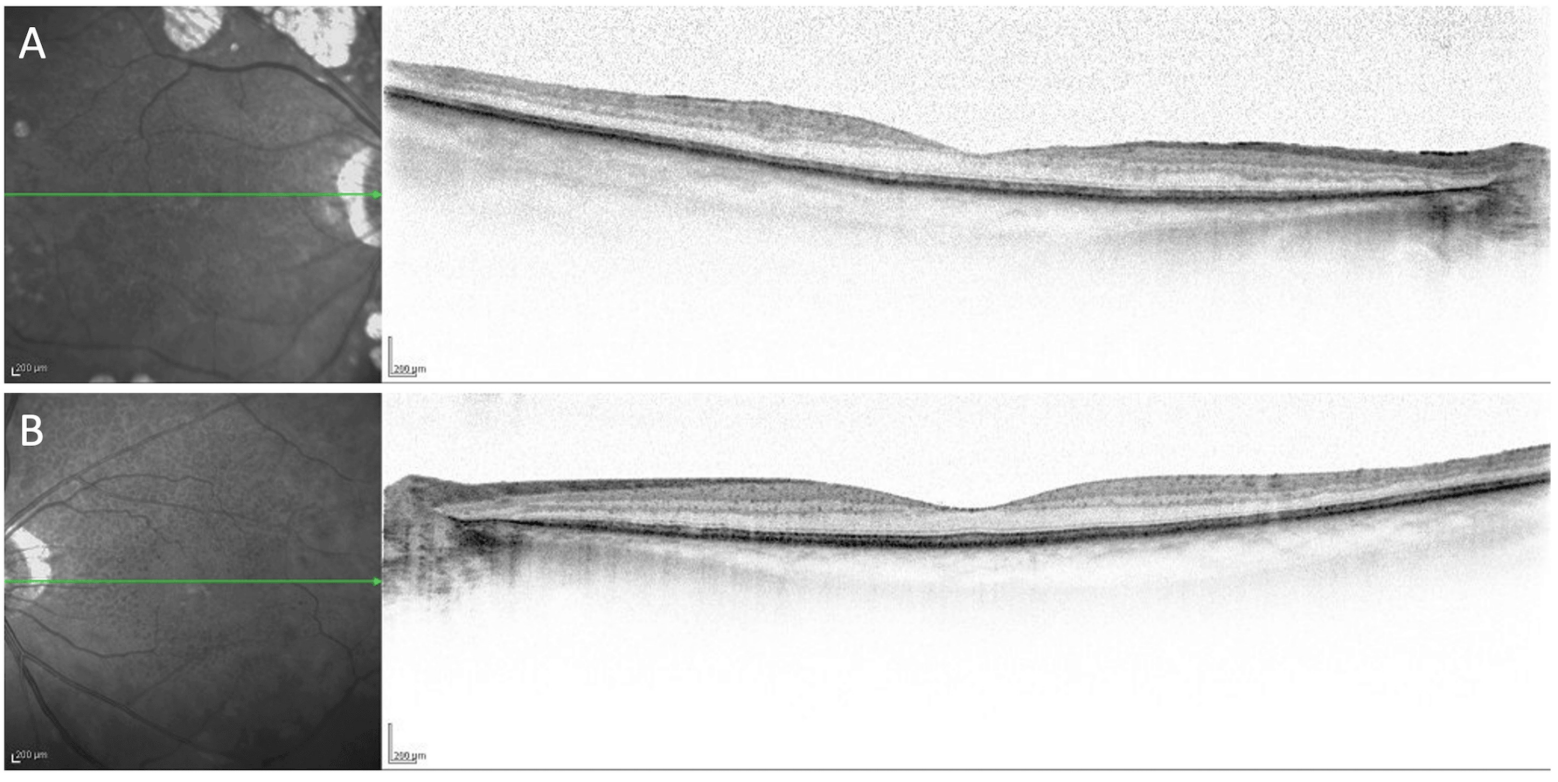
Images of spectral-domain optical coherence tomography A and B show spectral-domain optical coherence tomography images of the right and left eyes, respectively, taken at the current visit.

Optical coherence tomography at the current visit showed no retinal detachment and choroidal thickness was 186 and 196 μm in the right and left eyes, respectively (Figure 3A, 3B).

The patient had no history of ocular trauma or surgery and presented with no ocular symptoms. Ocular inflammation in the uveitis stage was not confirmed because the patient was unaware of the ocular symptoms and did not undergo an ophthalmic examination. However, the previous symptoms of meningitis and vertigo, as well as the current vitiligo of the skin and sunset glow fundus, suggested that the patient had Vogt-Koyanagi-Harada disease in the convalescent phase [8].

## Discussion

We report a case of VKH diagnosed based on vertigo, vitiligo, and sunset glow fundus. The absence of subjective ocular symptoms, such as decreased vision, as in this case, is uncommon.

There are no diagnostic serological markers for VKH. VKH is diagnosed on the basis of clinical symptoms and findings and proposed diagnostic criteria [8]. In the present case, vertigo, abdominal pain, keratic precipitates, vitiligo, and sunset glow fundus with nummular chorioretinal depigmented scars were noted. According to the revised diagnostic criteria for Vogt-Koyanagi-Harada disease [8], the patient was diagnosed with incomplete VKH. As the patient had keratic precipitates, sunset grow fundus, and vitiligo, the patient also met the classification criteria for late-stage VKH [9]. Analysis of 1147 patients with VKH from various geographic regions revealed that Hispanics and Asians are more likely to be diagnosed with VKH than with non-VKH than are individuals of other ethnicities, and that exudative retinal detachment in acute disease and sunset glow fundus in chronic disease are highly specific for patients with VKH [10]. Our patient presented with sunset glow fundus that had not been observed five years earlier, suggesting that potent choroiditis had developed, but the patient was unaware of any ocular symptoms such as vision loss.

The acute uveitic phase is characterized by bilateral blurring of vision due to uveitis, preceded by prodromal phase of meningitis symptoms (fever, headache, nausea, vomiting, and neck stiffness) and auditory manifestations (tinnitus, vertigo, and hearing loss) [4-6]. Fujiwara et al. investigated vestibular functions in patients with VKH disease and reported that the detection rate of abnormalities by various vestibular tests, including nystagmus test, caloric test, and vestibular evoked muscle potential test, was significantly higher than the detection rate of subjective symptoms such as vertigo [5]. Al Dousary reported that six of the nine patients with VKH who received early treatment regained their hearing, while three of those who received delayed treatment did not [6]. The present case was not treated due to lack of awareness of any ocular symptoms and delayed diagnosis of VKH. This results in residual vertigo.

In the chronic convalescent phase, the loss of melanin granules in the choroid and skin results in sunset glow fundus, poliosis, or vitiligo. In a study of 1147 patients with VKH, vitiligo reportedly occurred in 20% of cases [10]. Cases have also been reported in cases in which VKH was recognized because of the presence of vitiligo [11]. Generally, signs of ocular and skin depigmentation appear during convalescence. In the present case, skin depigmentation occurred approximately one month after the onset of vertigo and other symptoms. Yamamoto et al. compared VKH patients treated with systemic corticosteroids with untreated patients and reported that depigmentation occurred earlier in the untreated group (33.5 days) than in the systemically treated group (40.8 days) [12]. Vitiligo associated with VKH disease has been reported to occur mainly on the face, back (especially in children), and upper extremities (in adults), which differs from the common sites of non-VKH vitiligo, which mainly affects the face and lower extremities [7,13,14]. In this case, vitiligo occurred on the face and upper extremities, which is consistent with the predilection sites for vitiligo in VKH.

As in this case, Harada's disease is known to be occasionally associated with other autoimmune diseases such as type 1 diabetes and Graves' disease [15]. VKH has reportedly been associated with human leukocyte antigen (HLA) genotypes HLA-DRB1*0405, which are also disease susceptibility genes for both type 1 diabetes and Graves' disease in the Japanese population [16,17].

The treatment of VKH is usually high-dose intravenous corticosteroids for three days in the acute phase, followed by high-dose oral steroids. Early treatment with high-dose corticosteroids has been reported to be associated with a better prognosis in terms of visual acuity [18]. Delayed initiation of corticosteroid therapy in the acute phase increases the risk of recurrence in the chronic recurrent phase [2,3,19]. Fortunately, in the present case, there was no significant vision loss without treatment.

## Conclusions

Patients with VKH usually present with ocular symptoms such as decreased vision. In the present case, the patient was unaware of any ocular symptoms, and the diagnosis of VKH was based on the presence of vitiligo and sunset glow fundus. If sunset glow fundus is observed on fundus examination, the possibility of VKH should be considered and other systemic symptoms, including vertigo, hearing loss, headache, nausea, and vitiligo should be evaluated. As sunset glow fundus is a finding in the chronic recurrent phase, the patients should be carefully monitored for recurrence.
